# The Science and Social Necessity of Deceased Organ Donation

**DOI:** 10.5041/RMMJ.10048

**Published:** 2011-04-30

**Authors:** Francis L. Delmonico

**Affiliations:** Professor of Surgery, Harvard Medical School, Boston, USA; President-elect, The Transplantation Society, Montréal, Canada

**Keywords:** scientific, social, deceased, donation, achieve self-sufficiency

## Abstract

Successful deceased organ donation requires a reproducible – consistent (scientific) system that evaluates the potential for organ donation and determines objectively whether the national system is achieving its goals. The science of organ donation also pertains to the determination of death. We are a common humanity that dies similarly – a humanity whose ultimate criterion of life resides in the function of the human brain. The recent brain death law of Israel encouragingly enables a determination of death by the loss of neurologic function, but it has become complicated by a practice that may perpetuate societal misperceptions. As a result opportunities for deceased organ donation – to provide for Israelis in need of organ transplants – are being lost. A statured task force of society could be assembled to convey its support for deceased donation to influence society and resolve these misperceptions.

The World Health Organization is now calling for each member state to achieve a self-sufficiency in organ donation and transplantation “equitably meeting the transplantation needs of a given population using resources from within that population”. Patients should not be compelled to go to foreign countries for their organs. Israel has been a leader in the development of a model program intended to address transplant tourism. Insurance companies are no longer permitted to provide resources for Israelis to undergo illegal transplants in foreign destinations. The social necessity of a scientifically and medically applied system of deceased organ donation is now evident so that a sufficient number of organs can be available for patients from within the country where they reside.

## THE SCIENCE OF ORGAN DONATION

Deceased organ donation is a skilled discipline that brings an obvious benefit to donor families and transplant recipients. Deceased donation is a science by the collection of data and by developing best practices following an analysis of the data. Successful donation requires a reproducible, consistent (scientific) system that evaluates the potential for donation and determines objectively whether the system is achieving its goals. The extent and organization of that system are the key elements to maximizing the therapeutic potential of deceased organ donation.

The World Health Organization (WHO) has developed a critical pathway as a guide for each country as it strives to implement a deceased donor program and achieve national self-sufficiency.^1^ With national self-sufficiency, the organ donation and transplantation needs of a country are provided from resources within that country – not relying upon the population of another country (the poor and underprivileged) to be the source of its organs.

This WHO critical pathway for deceased donation is scientifically devised to capture categorically the procedural steps that enable a transition from a possible deceased donor, to an actual, and then a utilized donor by the recovery and transplantation of at least one organ from the donor.

The WHO critical pathway was drafted to be a tool for those who are responsible for assessing the opportunity of deceased organ donation in each hospital. This tool can be used retrospectively to assess performance and also prospectively to monitor deceased donor activity in a hospital, in a region, and ultimately within a country.

Each country is being called upon to integrate a program of deceased donation to its health care system as a societal responsibility to its people in need of organs. Resources must be provided in the health care budget and for the professionals involved in this effort. A successful worldwide donation experience necessitates ministry of health support, and governments should no longer presume that professional activity will be volunteered to achieve a sustainable program of deceased donation.

## ASSESSING DECEASED DONOR POTENTIAL

For each hospital the following data should be collected and analyzed categorically – consistent with the WHO critical pathway:

- How many patients are *possible donors* with a devastating brain injury in the intensive care unit (ICU)?
- How many are *potential donors –* anticipated to meet the criteria of a brain death diagnosis?
- How many patients die in the ICU with such a devastating brain injury and by what etiology – tumor, trauma, or stroke?
- How many patients are determined to be dead by neurologic criteria?
- How many patients would be *eligible donors –* defined as medically suitable and brain dead?
- How many eligible donors are identified and referred to the organ donation agency?
- How many are not? (How many were brain dead and not medically suitable – i.e. potential but not eligible?)
- How many eligible donors are *consented via family*?
- How many consented donors reach the operating room with the intent to donate or from whom one organ was recovered – i.e. an *actual donor* by the WHO critical pathway?
- How many actual donors become *utilized donors* in which at least one organ was transplanted?
- What is the rate of organs recovered and then transplanted per donor?

## REASONS WHY A POTENTIAL DONOR DOES NOT BECOME A UTILIZED DONOR

### SYSTEM

- Failure to identify/refer a potential or eligible donor
- Brain death diagnosis not confirmed (e.g. does not fulfill criteria) or completed (e.g. lack of technical resources or clinician to make diagnosis or perform confirmatory tests)
- Circulatory death not declared within the appropriate time frame
- Logistical problems (e.g. no recovery team)
- Lack of appropriate recipient (e.g. child, blood type, serology positive)

### DONOR/ORGAN

- Medical unsuitability (e.g. serology positive, neoplasia)
- Hemodynamic instability/unanticipated cardiac arrest
- Anatomical, histological, and/or functional abnormalities of organs
- Organs damaged during recovery
- Inadequate perfusion of organs or thrombosis

### PERMISSION

- Expressed intent of deceased not to be donor
- Relative’s refusal of permission for organ donation
- Refusal by coroner or other judicial officer to allow donation for forensic reasons

The reasons why a potential donor does not become a utilized donor can be assessed categorically with a strategic plan devised to overcome the obstacles that have been encountered.

## THE DETERMINATION OF DEATH

The science of organ donation also pertains to the determination of death. Death – scientifically and medically – is no different for the Hindu, Christian, Islamic, or Jewish heritage; we are a common humanity that dies similarly by a decomposition of our bodily organs within hours of our death. Death is indeed a process, but there is a finality that can be legally and medically determined; for again, we are common humanity whose ultimate criterion of life resides in the function of the human brain (responsible for our consciousness) and brain stem (responsible for our spontaneously breathing).

The function of the brain is not replaceable or transplantable in contrast to the function of other organs such as the heart, liver, and kidneys. The brain is accountable for our thought, our volition, our personality –ourselves. The irreversible loss of brain function is a clinical condition that is not restorable or resolvable. Thus, the concept of death by neurologic function or brain death affords an opportunity for deceased organ donation that is unique (versus heart or liver function) yet common to us all in a common humanity. That valid, scientific (consistently determined), medical, and now legal concept of death requires societal education to achieve its acceptance because the cultural objections, although understandable, are not sustainable by societal education regarding the medical (bodily decomposition) and scientific (irreplaceable brain function) realities of death.

## THE CONCEPT OF DEATH

The recent brain death law of Israel encouragingly enables a determination of death by the loss of neurologic function, but it has become complicated by a practice that may perpetuate societal misperceptions. Family objection to the discontinuation of mechanical ventilation despite a declaration of death by neurologic function may be understandable culturally, but it does not change outcome, and it impedes the opportunity for organ donation. In 2010, there were reportedly 29 patients with a devastating brain injury by stroke or trauma that fulfilled the criteria of death by assessment of neurologic function; however, the family was not accepting of the diagnosis. Public education would then seem to be important by an analysis of data as to those instances in which the family requested the continuation of the ventilator. What has happened to the patient following that family decision? Has the patient been restored to well-being, or was the finality of life confirmed by the determination of brain death? These data should be analyzed and reported through professional organizations and publicly in the media, about the reality and finality of brain death. Otherwise, opportunities for organ donation to provide for Israelis in need of transplants are being lost.

## NATIONAL SELF-SUFFICIENCY

The World Health Organization is now calling for each member state to achieve self-sufficiency in organ donation and transplantation,” equitably meeting the transplantation needs of a given population using resources from within that population”. The Declaration of Istanbul also calls upon each country to achieve self-sufficiency by providing a sufficient number of organs for residents in need from within the country or through regional co-operation. Co-operation between countries is consistent with national self-sufficiency, as long as the collaboration promotes a reciprocal provision of organs for each country. The transplantation of organs for patients travelling from outside a country or jurisdiction is only acceptable if it does not undermine the country’s ability to provide transplant services for its own population.

Transplant tourists leave their home country to undergo transplantation in foreign destinations – well known to the international community. For patients to acquire organs in foreign destinations is no longer acceptable to the rest of the world. Countries must now address the organ donation needs for their own people. Importantly, best care for Israelis or Americans or Canadians or patients from the Gulf countries or Asia is not achieved with programs of transplant tourism. Moreover, the main reason that such transplant care is being provided in a foreign destination is for commercial interests of the transplant centers and not for the ultimate provision of medical care. Once the transplant is performed, the transplant tourist returns home to use the resources of the home country to provide essential medical care. The medical literature is now replete with reports of the abuses and poor outcomes that occur in the setting of transplant tourism – for the transplant recipient and for the living donors.^2^–^5^

This principle of national self-sufficiency is now well recognized in Israel and in the rest of the world, highlighted by the professional community in the Declaration of Istanbul and by the adopted Resolution of the 63rd World Health Assembly – calling upon all jurisdictions to address their organ donation needs. Deceased organ donation becomes a social necessity for a community that requires crisis attention by government and society – no differently than other societal confrontations that threaten the viability of a people.

## COMMENDABLE STEPS BY ISRAEL TO CURTAIL TRANSPLANT TOURISM

Israel has been a leader in the development of a model program intended to address transplant commercialism and tourism. Insurance companies are no longer permitted to provide resources for Israelis to undergo illegal transplants in foreign destinations. The recent judicial ruling dismissing the litigation against the Phoenix Insurance Company that had denied coverage for a transplant in Kosovo is noteworthy. Israel has also developed a system to distribute deceased donor organs with a priority for those who are themselves accepting to be organ donors.^6^ Thoughtful leaders are calling upon Israeli society to support deceased organ donation by suggesting that if one anticipates that society will provide an organ for transplantation, one should be supportive of being an organ donor. Otherwise, it becomes difficult to reconcile a cultural basis for not consenting to deceased organ donation when members of the same community undergo organ transplantation from brain-dead donors in Colombia or Ecuador. A statured task force of society could be assembled to convey its support for deceased organ donation to influence the society as to the need for donor organs.

## THE SOCIAL NECESSITY OF DECEASED ORGAN DONATION

The organ donation crisis becomes apparent to Israelis and to the international community when the intent of a football star to be an organ donor is not fulfilled. His desire to provide his organs for Israeli patients in need was noble. Alternatively, for patients to be seemingly compelled to go to foreign countries for their organs is not ultimately acceptable either.

Will these organs be reliably available in foreign countries? There is a major change in the global environment today (compared to expectations only a few years ago) by the international resolutions to curtail transplant tourism. Again, the Resolution of the 63rd World Health Assembly in May 2010 and the Declaration of Istanbul are seminal documents that prohibit transplant commercialism, transplant tourism, and organ trafficking. As a result, the battle is now engaged in those countries to impede transplants for foreign patients.

Therefore, The Transplantation Society and the World Health Organization are supporting the robust program of deceased organ donation in Israel as a testimony and resolve of the Israeli people to provide for Israeli patients in need of organs. The science of organ donation as conducted by the WHO critical pathway provides a strategy to increase deceased organ donation – implementing best practices by accepted standards throughout the world. This WHO survey and critical pathway provides a tool for a consistent examination of the donor potential of Israel, conducted with scientific rigor. The scientific and social necessity for deceased donation is now urgent and clear.

## CONCLUSION

The Declaration of Istanbul and the Resolution of the 63rd World Health Assembly have become powerful forces to combat transplant commercialism, organ trafficking, and transplant tourism. That battle is now enjoined in Latin America – to prevent American insurance brokerages from setting up a program in several Latin American countries. It is enjoined in an effort, for example, to stop Canadians from purchasing organs in Pakistan and China and to curtail patients from the Gulf countries undergoing transplantation in the United States and in China. Colombia is now limiting transplant tourists from Japan, Israel, and elsewhere to constitute only 1% of the total transplant population. The attention of the international community may soon be directed to Ecuador, because of the reported intention to establish a system of transplant tourism there.

Arab Israelis that reportedly died following kidney transplantation in Egypt in 2010 underscore an important public message: countries must address their own organ donation and transplantation needs – for its people. Cultures should respect the wishes of individuals to be donors and conduct themselves consistently; if it is acceptable to undergo transplantation from a deceased donor in a foreign destination, then it should be acceptable (and encouraged) to undergo deceased donor transplantation in the country where the patient resides.
